# Face-Inversion Effect on Disgust Evoked by a Cluster of Dots

**DOI:** 10.1177/2041669518784960

**Published:** 2018-06-27

**Authors:** Manami Furuno, Yuri Sakurai, Shu Imaizumi, Shinichi Koyama

**Affiliations:** Graduate School of Engineering, Chiba University, Japan; Faculty of Art and Design, University of Tsukuba, Japan; Graduate School of Engineering, Chiba University, Japan; Graduate School of Arts and Sciences, University of Tokyo, Japan; Faculty of Art and Design, University of Tsukuba, Japan; Graduate School of Engineering, Chiba University, Japan

**Keywords:** trypophobia, face-inversion effect, disgust

## Abstract

A cluster of dots such as lotus seed pods evokes extremely strong disgust when it is placed on human and animal skins. However, few empirical studies have examined the role of the background image, such as skin, in the generation of disgust. In this study, we investigated whether the orientation of background faces influences the disgust evoked by the dot pattern. The participants were asked to evaluate disgust to an upright, inverted, or phase-scrambled face image with or without a cluster of dots on it and then complete a questionnaire measuring trypophobia proneness (Trypophobia Questionnaire). The results suggested that disgust was intensified by the background faces, especially by the upright faces. The intensification of disgust in the upright face was correlated positively with the Trypophobia Questionnaire scores. The results indicated a face-inversion effect on the disgust to the dot pattern, suggesting a significant role of the background image.

## Introduction

Disgust can be evoked by the observation of clusters of dots and small objects that should be innocuous. This disgust is referred to as trypophobia (Cole & Wilkins, 2013). Lotus seed pods are one of the most common trypophobic images (Cole & Wilkins, 2013; Le, Cole, & Wilkins, 2015). Interestingly, placing lotus seed pods on human skin increases disgust significantly. In Japan, some Internet artists (e.g., Hasumaru, 2018) have published images with lotus seed pods on human skin, and these are widely known as *HASU-COLLA* (*HASU* = lotus seed pods; *COLLA* [i.e., *collage*] = photomontage image).

Disgust to HASU-COLLA can be explained by two factors: dot patterns per se and the combination of dot patterns and the background image. Researchers first explained the disgust by the physical characteristics of the dots. Cole and Wilkins (2013) showed that trypophobic images have high-contrast energy at midrange spatial frequencies, and this tends to induce visual discomfort (Fernandez & Wilkins, 2008). Le et al. (2015) reported that the disgust is greater when the cluster size is larger and when the clusters consist of a mix of holes and bumps.

On the other hand, the disgust to the clusters was also explained by the combination of dots and background image (Kupfer & Le, 2018; Skaggs, 2014). Some researchers have hypothesized that disgust to the clusters of dots increases when they are presented on human skin because they remind us of scars and sores (Skaggs, 2014). This hypothesis was supported by a study in which viewers reported greater disgust when a cluster of dots was placed on faces rather than on stones (Furuno, Imaizumi, Maeda, Hibino, & Koyama, 2017). In addition, a study by Kupfer and Le (2018) reported that disgust to a cluster was stronger when the cluster consisted of disease-relevant elements such as circular rash marks on a chest than those consisting of disease-irrelevant elements such as drilled holes in a brick wall. Although the studies demonstrate the significant role of the background image on the disgust evoked by dot patterns, their visual stimuli were not physically controlled. They used different images for skin and nonskin conditions, and physical properties of the images such as luminance, color, and spatial frequency spectrum were different between the two conditions.

In this study, using physically controlled visual stimuli, we tested the hypothesis that face-likeness or skin-likeness of the background image significantly increases disgust evoked by a cluster of dots. Here, we compared disgust to the same dot pattern on upright and inverted faces. We used faces for the background image because we can change emotional impacts of the stimuli by simply changing their orientations, as indicated by the Thatcher illusion (Thompson, 1980). This effect is known as the face-inversion effect (Valentine, 1988). We can recognize emotional expressions more rapidly in upright faces than in inverted faces (Eimer & Holmes, 2002). Face-inversion effect can occur not only on the behavior level but also on the neural response level (Ashley, Vuilleumier, & Swick, 2004). Disgust to the cluster of dots will be stronger in the upright faces than in the inverted faces because the dots will be perceived more as part of the face in the upright face and look more like disease-relevant than in the inverted face. Moreover, we also compared disgust to the same dot pattern on the upright and phase-scrambled faces. Because the phase-scrambled faces were generated just by shifting the spatial frequency spectrum of the upright faces, both faces had the same spatial frequency properties. If the disgust to the dot pattern is stronger in the upright faces than in the phase-scrambled faces, it would suggest that high-level cognitive processing, like disease-avoidance, is involved in the induction of disgust.

We also examined whether trypophobia proneness correlates with the face-inversion effect. Studies have shown individual differences in the sensitivity to a cluster of dots (Cole & Wilkins, 2013; Imaizumi, Furuno, Hibino, & Koyama, 2016b). The individual differences in the sensitivity to lotus seed pods have been partly explained by general disgust sensitivity and empathic traits (Wada, 2016). However, it is unknown whether trypophobia proneness is amplified in a certain stimulus or combination of dots and background.

## Method

### Participants

Twenty-four adults (10 females; mean age 22.2 ± 1.37 years) participated in this study. Written informed consent was obtained from each participant. The experiments were conducted according to the principles of the Declaration of Helsinki and approved by the Ethics Committee of the Graduate School of Engineering, Chiba University (Approval Number 28-06).

### Apparatus and Stimuli

**Figure 1.**
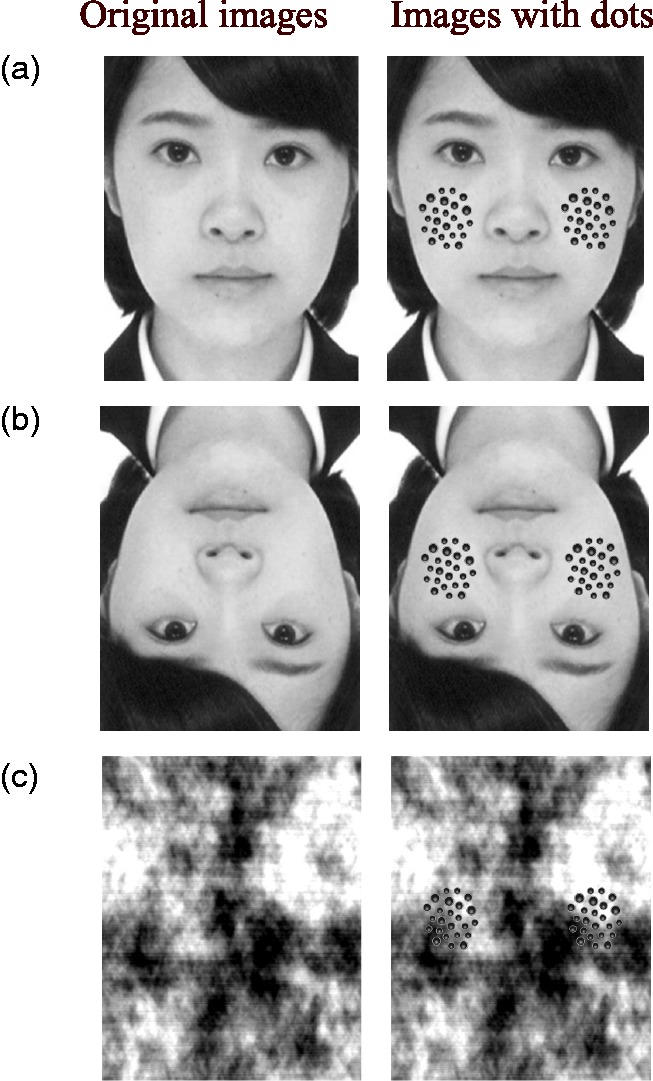
(a) Upright, (b) inverted, and (c) scrambled original images (left) and images with clusters of dots that were placed on the original images (right).

We used front face images of four Japanese females and four Japanese males. We used two female images and two male images from Japanese and Caucasian Facial Expressions of Emotion (Biehl et al., 1997) and two female images and two male images from the Sozai-jiten Picture Database (Imagenavi, 2018; Figure 1(a) left). The images were grayscaled, and the average luminance of the images was set to approximately 19.6 cd/m^2^ in all images. As control images, we also used spatially inverted images of the original images and phase-scrambled images made with a MATLAB code (Figure 1(b) left and (c) left). Because we only inverted or phase-scrambled the original images, the original, inverted, and phase-scrambled images had the same luminance and spatial frequency spectrum. We made another 24 images by placing a cluster of black dots on both left and right sides of the picture at the vertical midline in each type of stimulus (original, inverted, and scrambled). In total, there were 48 images. The stimulus size was 18.0° × 13.7° (680 × 520 pixels). The viewing distance was 57 cm, so 1 cm was almost equivalent to 1° visual angle.

In the images with a cluster of dots, a cluster of 42 dots on each side, 84 dots in total, was placed on the original image. Among them, the dot size of the 24 dots was 0.3° in diameter, and the dot size of the remaining 18 dots was 0.4° in diameter. We placed 84 dots on the original images based on the study by Le et al. (2015), which suggested that stronger disgust is invoked when the number of dots is between 64 and 256, rather than 16. We also avoided using too many dots in order to see the background face clearly. The cluster consisted of a spatial frequency between 2.7 and 18.5 cycles/degree. The dots were placed almost evenly in an oval area measuring 3.9° × 3.5°. The color of the dots was black and gray with circular gradation. The dots were drawn using Adobe Photoshop CS6. In the experiment, the stimuli were presented in a darkened room. Participants rested on a chin rest and viewed the stimuli. Stimuli were presented on a 24-in. monitor (ASUS VG248QE) with a resolution of 1,920 × 1,080 pixels. We controlled the stimuli presentation by using Hot Soup Processor 3.4 (ONION Software, 2016).

### Procedure

Participants first took part in a disgust evaluation experiment. At the beginning of the experiment, there were two practice trials. In the practice trials, a fixation point was first presented in the center of the monitor for 0.75 seconds on the 24-in. monitor. Next, a stimulus that was irrelevant to the main trials (e.g., a gray square) was presented for 10 seconds. Finally, these instructions were presented: “Please push the number to indicate how you felt when you viewed the above image.” The participants were asked to rate the image using a 9-point scale ranging from 1 (*not unpleasant at all*) to 9 (*extremely unpleasant*) using the numeric keypad.

After the practice trials, there were 48 main trials. In each trial, one of the 48 images (consisting of eight upright/inverted/scrambled face pictures with and without a cluster of dots) was presented in random order. A fixation point was presented for 0.75 seconds, and an image was presented for 10 seconds. Participants were asked to rate the image using the 9-point scale ranging from 1 (*not unpleasant at all*) to 9 (*extremely unpleasant*). There was an intertrial interval of 0.5 seconds for each trial during the main task.

After the experiment, participants completed the Japanese version of the Trypophobia Questionnaire (TQ: Le et al., 2015), that is, TQ-J (Imaizumi, Furuno, Hibino, & Koyama, 2016a), which measures trypophobia proneness. At the beginning of the TQ-J, two trypophobic images, a picture of a lotus seed pod and that of a honeycomb, were presented sequentially on the 24-in. monitor. The participants viewed the images and reported their impression of the images. They received 6 emotional items (e.g., “Feel anxious, full of dread or fearful” and “Feel aversion, disgust or repulsion”), 11 somatic items (e.g., “Feel skin crawl,” and “Vomit”), and 2 foil items (e.g., “Feel at peace” and “Want to laugh”). They rated these based on their agreement with the items using a 5-point scale ranging from 1 (*not at all*) to 5 (*extremely*). Responses were summed up to produce a total score of trypophobia proneness. Based on the screening criteria in the original TQ (Le et al., 2015), we excluded participants who rated the foil items of the TQ-J as other than “not at all.” The final analyses included a total of 21 participants (10 females; mean age 22.0 ±1.34 years).

## Results

**Figure 2.**
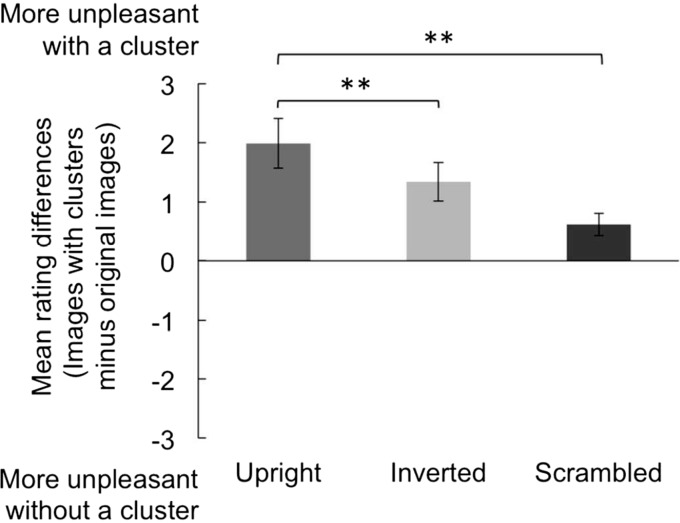
Average scores of differential values of the original images and those with clusters in upright/inverted/scrambled images. Error bars denote standard errors of the mean. **p < .01.

First, we analyzed whether the increase in disgust was observed by placing the clusters on the upright/inverted/scrambled images. To calculate the increasing quantity of disgust, we subtracted rating scores for the original upright/inverted/scrambled faces from the rating scores for the upright/inverted/scrambled images with dots. Figure 2 shows the mean rating differences of disgust scores for upright/inverted/scrambled with clusters and those without clusters. One-sample two-tailed *t* tests revealed that the differences in the disgust scores between the original images and the images with dots were significantly greater than 0 for all types of faces, upright: *t*(20) = 4.69, *p* < .001, *r* = .72; inverted: *t*(20) = 4.12, *p* < .01, *r* = .68; scrambled: *t*(20) = 3.31, *p* < .01, *r* = .60. A series of one-way repeated measures analyses of variance on the differences showed a significant main effect for face, *F*(2, 40) = 11.72, *p* < .01, η^2 ^= 0.37. Multiple comparisons using the Bonferroni method indicated that the difference between the original images and the images with dots was significantly greater in the upright faces than in the inverted faces and scrambled faces (*p* < .05). Multiple comparisons by the Holm method also indicated that the difference was significantly greater in the inverted faces than in the scrambled faces (*p* < .05), although the Bonferroni method indicated that the difference was marginal (*p* = .06).

The average disgust ratings were 4.61 (standard error [*SE*] = 0.25) for the original upright faces, 5.28 (*SE* = 0.20) for the original inverted faces, and 5.03 (*SE* = 0.21) for the original scrambled faces. The average disgust ratings were 6.48 (*SE* = 0.24) for the upright faces with dots, 6.74 (*SE* = 0.20) for the inverted faces with dots, and 5.65 (*SE* = 0.19) for the scrambled faces with dots.

**Figure 3.**
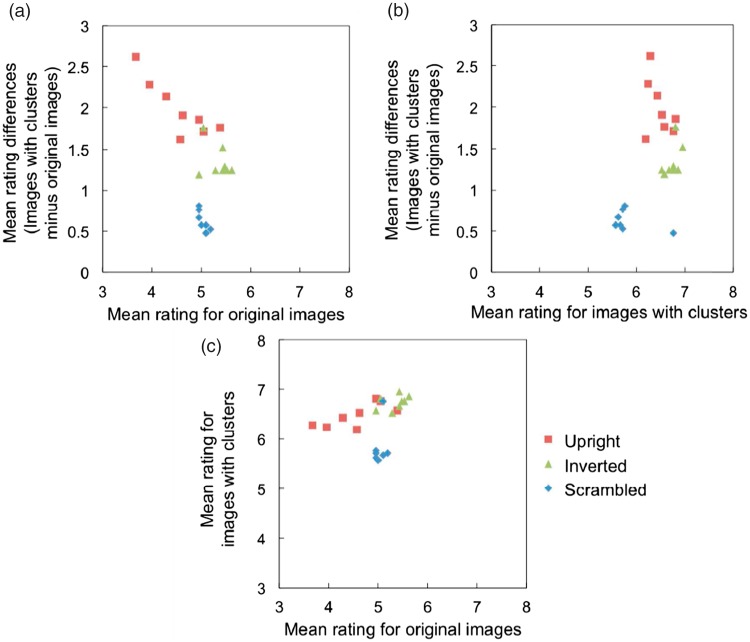
Scatter plots for (a) mean ratings for the original images and mean rating differences, (b) mean ratings for images with clusters and mean rating differences, and (c) mean ratings for original images and images with clusters. Red squares denote upright faces, green triangles denote inverted faces, and the blue rhombuses denote scrambled faces.

Figure 3 shows results of the correlation analysis between the difference scores and raw scores (from original images) for each image. The results showed a significant negative correlation for the upright faces, *r*(6) = −.86, *p* < .01, although there was no significant correlation for the inverted faces, *r*(6) = −.33, *p* = .43. There was a significant negative correlation for phase-scrambled faces; however, the correlation was weaker than that for the upright faces, *r*(6) = −.76, *p* < .05.

Second, we analyzed whether there was a correlation between TQ-J score and disgust ratings. The analysis indicated that there was a significant positive correlation, *r*(19) = .55, *p* < .01, between the TQ-J scores (*M* = 31.1, *SE* = 2.94) and the average disgust ratings of all 24 images with clusters (*M* = 6.29, *SE* = 0.18) from each participant. The results also indicated a significant positive correlation, *r*(19) = .75, *p* < .01, between the TQ-J scores and the difference between the average disgust ratings of the 24 images with dots and those of the 24 original images (*M* = 1.32, *SE* = 0.28) from each participant. We also performed correlation analyses between the TQ-J scores and the difference between the average disgust ratings of the eight images with dots and those of either original images for each background image (upright/inverted/scrambled) from each participant. The results indicated significant positive correlations between the TQ-J scores and the difference for all background images, but the correlation was stronger for the upright and inverted faces than for the scrambled faces, upright: *r*(19) = .73, *p* < .01; inverted: *r*(19) = .73, *p* < .01; scrambled: *r*(19) = .49, *p* < .05.

## Discussion

We investigated the influence of the background image on disgust evoked by clusters of dots using the face-inversion effect. The experimental procedures helped us to systematically change the face-likeness and skin-likeness of the background image, controlling for the physical features between the conditions.

Our results indicate that the background image influenced the disgust to the clusters. The disgust to the clusters was the strongest when they were placed on the upright faces, in which faces looked the most face-like and the skin looked the most skin-like. The results support our hypothesis that disgust to the clusters depends on perceived face-likeness and skin-likeness of the background image. Further, our results can be at least partly explained by the hypothesis that the disgust to the clusters of dots is caused by the reminder of disease and contamination (Furuno et al., 2017; Kupfer & Le, 2018; Rozin, Haidt, & McCauley, 2008; Skaggs, 2014; Wada, 2012, 2016; Yamada & Sasaki, 2017). Our results were consistent with the previous study suggesting that disgust to the clusters on the skin was stronger than dots on the nonskin objects, such as wood grain (Wada, 2016) and stones (Furuno et al., 2017). We did not see any signs of ceiling effect: The average disgust rating to the upright and inverted faces, with and without dots, was around 5 to 6 points of the 9 points.

Face-inversion effect in the experiment suggests that dots were perceived as a part of the face, at least in the upright face. Participants felt less disgust to the inverted face perhaps because face inversion causes a disruption or delay in the initial analysis of emotional facial expression (Eimer & Holmes, 2002). The disruption or delay may result in the weaker reminder of scars and sores. The disgust to the clusters was stronger in the inverted faces than the scrambled faces perhaps because the inverted faces looked more like a face than the scrambled faces. However, local luminance contrast may also partly contribute to the disgust ratings, that is, disgust ratings for the scrambled faces were smaller than those for the upright and inverted faces partly because the local luminance contrast around the dots was smaller in the scrambled faces than in the other faces. As physical characteristics of images are an important factor for trypophobia, we should consider this point as a limitation of this study.

The results from the correlation analysis showed that participants did not simply add the constant amounts of scores to the image with a cluster. For the upright faces, there was a negative correlation between the ratings of the original image and the difference (rating of the original image subtracted from the rating of the image with a cluster), suggesting that participants added less scores when the cluster was placed on more unpleasant faces. On the other hand, for the inverted faces, there was no correlation between them. For the scrambled faces, there was a correlation between them; however, the correlation was weaker than for the upright faces. This suggests that participants’ response depends on the actual characteristics of the stimuli, rather than their expectation of the “correct answers.”

The results also indicated a strong correlation between the intensification of disgust to the clusters by the background of the face and the proneness to the trypophobic images. The TQ-J scores were correlated more strongly with the difference between the disgust ratings of the images with dots and the original images, *r*(19) = .75, *p* < .01, than the disgust to the images with dots per se, *r*(19) = .55, *p* < .01. The TQ, which measures trypophobia proneness, includes items related to the skin such as “Feel skin crawl” and “Have goosebumps” (Le et al., 2015). One possible hypothesis is that the intensity of disgust to the clusters by the background image is mediated by the proneness to trypophobic images. In conclusion, our study demonstrated significant effects of the background image on trypophobia. Our results from the experiment and the TQ-J together suggest that disgust to a cluster of dots increases for the upright faces and that the effect is stronger in those individuals who are prone to trypophobic images.
